# Aqueous-Cellulose-Solvent-Derived Changes in Cellulose Nanocrystal Structure and Reinforcing Effects

**DOI:** 10.3390/polym15143030

**Published:** 2023-07-13

**Authors:** Yuqi Tong, Shuting Huang, Xianjun Meng, Yixiang Wang

**Affiliations:** 1 Department of Food Science and Agricultural Chemistry, McGill University, Ste Anne de Bellevue, QC H9X 3V9, Canada; yuqi.tong@mail.mcgill.ca (Y.T.); shuting.huang@mail.mcgill.ca (S.H.); 2 Department of Food Science and Engineering, Shenyang Agricultural University, No. 120 Dongling St., Shenhe District, Shenyang 110866, China

**Keywords:** cellulose nanocrystals, cellulose solvents, crystal structure, reinforcing effect

## Abstract

Cellulose nanocrystals as reinforcing agents have received considerable interest, and their dimension mainly depends on the original sources of cellulose. We intend to manually modulate the morphology of cellulose nanocrystals by treating them with cellulose solvents so that we can explore their reinforcing capacity. In this work, waste cotton fabric was processed in two aqueous solvents (a sulfuric acid aqueous solution and a NaOH/urea aqueous solution), and the regenerated cellulose was used to produce cellulose nanocrystals using acid hydrolysis. The results revealed that the nanocrystals (RCNC-H) obtained after the treatment in sulfuric acid had a hybrid crystalline structure and a needle-like shape with an aspect ratio of about 15.2, while cotton fabric was completely dissolved in the NaOH/urea aqueous solution, and the regenerated nanocrystals (RCNC-N) displayed a typical crystalline form of cellulose II with a higher crystallinity and a shorter rod-like shape with an aspect ratio of about 6.3. The reinforcing effects of RCNC-H and RCNC-N were evaluated using polyvinyl alcohol (PVA) films as a model, where the addition of RCNC-H resulted in a relatively better tensile strength and oxygen barrier property, and the PVA/RCNC-N films had a slightly lower water vapor permeability. Therefore, this work suggests a new possibility for altering the naturally formed nanostructure of cellulose for different applications.

## 1. Introduction

Cellulose is the most abundant, readily available, and renewable natural polymer on Earth [1]. As an emerging class of nanomaterials, cellulose nanocrystals (CNCs) have received considerable interest over the past decades because of their unique properties such as biodegradability [2], biocompatibility [3], and reinforcing capacity [4]. CNCs can be extracted from many resources, such as plants, animals, bacteria, and even waste biomass [5,6,7], by using acid hydrolysis [8], chemical oxidation [9], mechanical methods with pretreatments [10], and a biological enzyme method [11]. Among them, sulfuric acid hydrolysis is the most traditional method for extracting CNCs [12]. The physical features such as the dimension and crystallinity index of CNCs mainly depend on their original sources [13]. For example, Zhong et al. extracted CNCs from indigo-dyed denim fabric using sulfuric acid hydrolysis, and the resultant CNCs showed a similar yield and morphology to those from bleached cotton but a higher crystallinity and thermal stability than wood CNCs [14]. In another comparative study, rod-like CNCs obtained from bleached cotton via sulfuric acid hydrolysis were longer and wider than flax-derived CNCs, and their aggregation tendency in the aqueous suspension differed from each other [15]. However, the efforts on modulating these naturally formed CNCs are limited.

Cellulose is insoluble in most commonly used solvents because of the inter- and intra-molecular hydrogen bonds and hydrophobic interactions [16]. Several solvent systems of cellulose have been reported (for instance, N-methylmorpholine-N-oxide (NMMO), LiCl/N,N-dimethylacetamide (DMAc), ionic liquids (ILs), deep eutectic solvents, NaOH/urea aqueous solution, and sulfuric acid aqueous solution) [17,18]. During the dissolution, the interactions between the cellulose molecules are “switched off” so that they can rearrange into new structures in the coagulation bath. The characteristics of the regenerated cellulose vary according to the solvent systems and the regeneration conditions. For example, after dissolving cellulose in ILs, using water as an anti-solvent for regeneration might generate significant crystallographic planes of cellulose II compared with other anti-solvents such as ethanol, acetonitrile, and so on [19]. In another research study, lyocell fibers were compared with viscose and modal cellulosic fibers where lyocell was mainly made up of cellulose crystalline II and amorphous cellulose and had a high level of crystallinity and thermal stability. Thus, it will be interesting to use this strategy to generate CNCs with structures that are different from their original forms.

With the consideration of sustainability, “green” aqueous solvents of cellulose were selected in this work to treat waste cotton fabrics, and the regenerated cellulose was hydrolyzed to prepare CNCs. The effect of the aqueous solvent treatment on the morphology and structure of CNCs was studied. Moreover, polyvinyl alcohol (PVA) films were employed as a model to evaluate the optical, mechanical, and barrier properties of CNC-incorporated composite films.

## 2. Materials and Methods

### 2.1. Materials

Waste cotton fabrics (100% cotton) were kindly provided by Renaissance (Montreal, QC, Canada). Sulfuric acid (H_2_SO_4_, 95.0–98.0%), sodium hydroxide (NaOH, ≥98%), sodium bromide (NaBr), urea, and polyvinyl alcohol (PVA) were purchased from Sigma-Aldrich (Oakville, ON, Canada) and used without further treatment.

### 2.2. Preparation of RC Samples

Cellulose dissolution and regeneration using H_2_SO_4_ and NaOH/urea aqueous solvents were performed according to the methods developed by Zhou et al. with some modifications [20]. Waste cotton fabrics were pre-treated with 20 wt% H_2_SO_4_ for 72 h. For RC from the H_2_SO_4_ solvent, 0.6 g of pre-treated fabrics was added into 20 mL of 64 wt% H_2_SO_4_ that was pre-cooled to −20 °C and stirred at 600 rpm for 10 min in an ice bath. The obtained solutions were centrifuged at 6500 rpm for 5 min to remove bubbles and then spread onto a glass plate and regenerated in a coagulation bath containing 10% (*w*/*v*) NaOH aqueous solution for 15 min. The regenerated films were washed with distilled water and air-dried for further use.

For RC from the NaOH/urea solvent, 0.83 g of pre-treated fabrics was mixed with 20 mL of 7% NaOH/12% urea aqueous solution and frozen overnight under −20 °C. After thawing, the samples were stirred at 2000 rpm for 8 min to obtain transparent cellulose solutions. The solutions were centrifuged at 6500 rpm for 5 min, spread on a glass plate, and coagulated in a 5% (*w*/*v*) H_2_SO_4_ aqueous solution for 5 min, followed by washing with water and drying in air. Cellulose regenerated from H_2_SO_4_ and NaOH/urea aqueous solvents were coded as RC-H and RC-N, respectively.

### 2.3. Extraction and Characterization of RCNCs

The CNCs were extracted from RC-H and RC-N using acid hydrolysis, as described by Bras et al. [21]. Generally, 1 g of RC was mixed with 20 g H_2_SO_4_ (64 wt%) and stirred for 1 h at 45 °C. Afterwards, the reaction was quenched by adding distilled water. In order to remove the excess acid, the suspension was washed with distilled water several times and then dialyzed against distilled water until the pH was stable. The CNCs extracted from RC-H and RC-N were coded as RCNC-H and RCNC-N, respectively. After freeze-drying, the yields (based on the dry mass of waste cotton fabrics) of RCNC-H and RCNC-N were calculated as 27.9 ± 4.8% and 24.4 ± 3.2%, respectively.

RCNC-H and RCNC-N suspensions were diluted to 0.05%, and their morphology was observed by using a Talos F200X G2 TEM (Thermo Fisher Scientific Inc., Ottawa, ON, Canada) at 200 kV. ImageJ 1.53t software (National Institute of Health, Bethesda, MD, USA) was used to measure the dimensions of RCNCs.

The zeta potential of RCNC-H and RCNC-N was measured in triplicate using a Zeta PALS Analyzer (Brookhaven Instruments Corp., Holtsville, NY, USA) based on electrophoretic light scattering at room temperature.

The FT-IR spectra of RC-H, RC-N, RCNC-H, and RCNC-N were obtained using a Varian Excalibur 3100 FT-IR spectrometer (Varian, Palo Alto, CA, USA) equipped with an attenuated total reflectance (ATR) accessory (Specac Ltd., Orpington, UK). All samples were scanned within the range of 650–4000 cm^−1^, and the spectra were collected as an average of 72 scans at a resolution of 2 cm^−1^.

The XRD patterns of RC-H, RC-N, RCNC-H, and RCNC-N were collected using an Empyrean 3 X-ray diffractometer (Malvern Panalytical Ltd., Malvern, UK) in a Bragg–Brentano configuration where CuKα target radiation between 4 and 40° was used. The crystallinity index (CrI) was determined using the peak height method [22]:

CrI = (I_ac_ − I_am_)/I_ac_ × 100% (1)

where I_ac_ represents the maximum intensity of the peak (200) crystallographic plane at around 2θ = 22.2° for cellulose I and the peak (020) at around 2θ = 20.4° for cellulose II. I_am_ represents the lowest intensity between planes (110) and (200) or (110) and (020), which is the intensity of diffraction attributed to amorphous cellulose.

The structure of RCNC-H and RCNC-N was examined using solid-state NMR on a Varian VNMRS spectrometer (Varian, Palo Alto, CA, USA) equipped with a 7.5 mm Varian Chemagnetics double-resonance probe operating at 399.9 MHz for 1 h and 100.5 MHz for ^13^C. The spinning speed was set at 3 kHz, and TOSS was used to suppress spinning sidebands, recording 12,288 scans with a contact time and recycle delay of 2 ms and 4 s, respectively. The crystallinity index (CrI_NMR_) was calculated using the following equation [23]:

CrI_NMR_(%) = Ac/(Ac + Aa) × 100% (2)

where Ac and Aa represent the fitted signal intensity of both the crystalline region and the amorphous region from the whole of the spectral C4 region, respectively.

### 2.4. Preparation and Characterization of PVA/RCNC Composite Films

The PVA/RCNC-H and PVA/RCNC-N films were prepared according to the previous method with some modifications [24]. Briefly, 1 g of PVA, together with 0, 1%, 5%, and 10% of RCNC-H or RCNC-N (based on the dry weight of PVA), was added in 20 g of water and stirred at 110 °C for 1 h. The suspensions were continuously stirred for another 24 h at room temperature and then cast and dried in a mold. The composite films were coded as NEAT, 1H, 5H, 10H, 1N, 5N, and 10N, corresponding to the content and type of RCNCs and stored at 58% relative humidity (RH) before the tests to simulate the environment’s humidity [25].

The optical transmittance of the NEAT, 10H, and 10N films was measured by a DU 800 UV−V is spectrophotometer (Beckman Coulter, Brea, CA, USA). The spectra were recorded with air as the background over a wavelength range from 200 to 800 nm.

The mechanical properties of PVA/RCNC composite films were tested according to the ASTM D882 standard using an eXpert 7601 single column testing machine (ADMET, Norwood, MA, USA) at room temperature. The deformation rate was 20 mm/min, and the initial grip separation distance was 20 mm. The dimension of the film specimens was 50 mm × 10 mm (length × width). The thickness of the films was determined by using a traceable digital caliper (Thermo Fisher Scientific Inc., Ottawa, ON, Canada), which was about 0.15 ± 0.02 mm.

The WVP of PVA/RCNC composite films was tested according to Koosha et al. with some modifications [26]. The films were sealed over a glass jar containing anhydrous calcium chloride. The glass jars were then placed in a desiccator containing distilled water at room temperature. The jars were weighed regularly to determine the mass increment on an analytical balance for 5 days. The WVP values were calculated based on the following equation:

WVP = ΔM × L/(A × t × ΔP)(3)

where ΔM is the mass increment of the glass jar (g), L is the film thickness (m), A is the permeation area (m^2^), t is the time (h), and ΔP is the partial pressure difference existing between the two sides of the film sample (Pa).

The OTR values were tested at 60% RH and 23 °C using the Ox-tran permeability tester (model 2/22, Mocon, Inc., Brooklyn Park, MN, USA).

### 2.5. Statistical Analysis

All experimental data are presented as mean ± standard deviation (SD), and the experiments were conducted in triplicate. Statistical analysis was performed using analysis of variance (ANOVA) followed by LSD (*p* < 0.05).

## 3. Results and Discussion

### 3.1. RCNCs Morphology and Surface Charge

**Figure 1:**
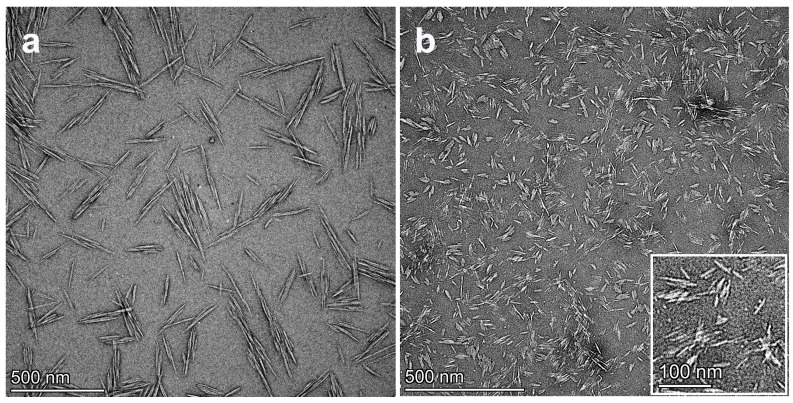
TEM images of (**a**) RCNC-H and (**b**) RCNC-N.

The transmission electron microscopy (TEM) images of RCNC-H and RCNC-N are shown in Figure 1. It was obvious that RCNC-H and RCNC-N had different morphologies. In particular, RCNC-H presented a needle-like shape with a length of 249.5 ± 85.6 nm and a diameter of 18.4 ± 7.8 nm, contributing to an aspect ratio of 15.2 ± 5.9 while RCNC-N showed a similar diameter of 15.1 ± 10.5 nm but a much shorter length of 87.9 ± 58.5 nm and a smaller aspect ratio of 6.3 ± 3.0. According to our previous work [20], the length and diameter of CNCs directly obtained from waste cotton fabrics using sulfuric acid hydrolysis were 111.8 ± 38.7 nm and 11.2 ± 2.3 nm, respectively, and their aspect ratio was 10.0 ± 3.4. These values were smaller than those of RCNC-H but larger than the dimension of RCNC-N. This suggested that the treatments of aqueous cellulose solvents did have an impact on the size of the nanocrystals. Both RCNC-H and RCNC-N still had a rod-like structure, which was important to their reinforcing effect [21,27,28].

The stability of the CNC suspension can affect the dispersion of CNCs in the material matrix and is related to the surface charge of CNCs. A nanocellulose suspension is considered stable when the absolute zeta potential value is greater than 30 mV. With this amount of surface charge, sufficient repulsive force exists among CNC particles to prevent aggregation [24]. In this study, both RCNC-H and RCNC-N exhibited high zeta potential values of −48.2 mV and −51.3 mV, respectively, which were higher than CNCs prepared from cotton fiber (a zeta potential value of −25.4 mV) [25]. This explained the well-dispersed CNC particles during TEM observation, and a good distribution of RCNC in the composite materials could be expected [26].

### 3.2. RCNCs Structure

**Figure 2:**
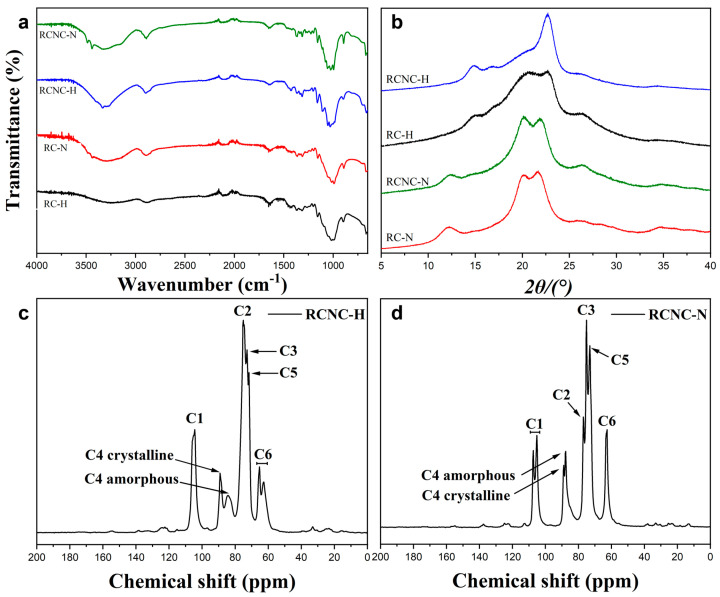
(**a**) FTIR spectra and (**b**) XRD patterns of RC-H, RC-N, RCNC-H, and RCNC-N. NMR spectra of (**c**) RCNC-H and (**d**) RCNC-N.

The FTIR spectra of the regenerated cellulose (RC-H and RC-N) and the corresponding CNCs (RCNC-H and RCNC-N) are shown in Figure 2a. All patterns had a broad band within the range of 3700–3000 cm^−1^ with respect to O–H stretching that presents considerable information concerning the hydrogen bonds. Along with the C-H and C-O stretching vibrations at around 2900 cm^−1^ and 1063 cm^−1^, the results confirmed that the cellulose supramolecular structure was retained after dissolution, regeneration, and acid hydrolysis. Although most characteristic peaks of RCNC-H were in accordance with RC-H, the C-H stretching band in RC-H was found between 2901 cm^−1^ and 2891 cm^−1^ (2896 cm^−1^), which suggested the presence of hybrid cellulose I and II structures [29]. Compared to the samples treated by H_2_SO_4_, the spectra of RC-N and RCNC-N showed two additional distinctive peaks at 3444 cm^−1^ and 3488 cm^−1^, which corresponded to the intramolecular hydrogen bonds of cellulose II. The stretching vibration peak of C-H in methyl and methylene groups in RC-N and RCNC-N shifted from 2901 cm^−1^ to 2891 cm^−1^, and the symmetric –CH_2_ bending peak at 1428 cm^−1^ in RCNC-H was observed to have a lower wave number at 1420 cm^−1^, representing the different CH_2_OH conformations of trans–gauche and gauche–trans. Moreover, the band at 710 cm^−1^ assigned to C-O-H out-of-plane bending in RCNC-H decreased in RC-N and RCNC-N. The asymmetric stretching vibration peak of C-O-C at 897 cm^−1^ in RCNC-H was detected at 893 cm^−1^, and the peak at 1110 cm^−1^ in RCNC-H contributed to the planar stretching vibration of the ring skeleton that was absent in RC-N and RCNC-N. These results demonstrated that RCNC-H mainly comprised cellulose I, and the crystalline structure of RC-N and RCNC-N comprised cellulose II [30].

Figure 2b displays the XRD patterns of the regenerated cellulose and CNCs. As is well-known, the XRD pattern of cotton cellulose is a typical cellulose I lattice [31]. After dissolving in NaOH/urea aqueous solution, RC-N showed characteristic peaks at 2θ = 12.2°, 20.1°, and 21.8°, corresponding to the (1$\bar{1}$0), (110), and (020) crystal planes, respectively, and suggesting a crystalline form of cellulose II [32]. This was because the cellulose chains rearranged into the stable anti-parallel structure when the hydroxide ions in the NaOH/urea solution were removed from the lattice of the cellulose matrix [33]. After acid hydrolysis, RCNC-N remained the typical cellulose II. Interestingly, the crystalline pattern change in RC-H was different. There was a doublet peak at 2θ = 20.5° and 22.6° and a less prominent diffraction peak at 2θ = 14.9°, indicating the existence of both cellulose I and cellulose II [29]. It was reported that the dissolution of cellulose in sulfuric acid is less fast at −20 °C [34], so some original crystalline structures in waste cotton fabrics remained in the solution, and cellulose II was formed from the dissolved portion during the regeneration. The pattern of RCNC-H showed two obvious diffraction peaks at 2θ = 14.9° and 22.6° and a small peak at 2θ = 16.6°, corresponding to the crystal planes of (1$\bar{1}$0), (200), and (110), respectively. The side peak near 2θ = 22.6° still existed, which indicated the dominant form of cellulose I in RCNC-H and the sustained cellulose II after acid hydrolysis [31,35]. It could be suspected that the newly formed cellulose II crystals during the regeneration would combine with the original crystalline structure and protect it when treated with acid hydrolysis, so the length and diameter of RCNC-H were greater than those of CNCs directly obtained from waste cotton fabrics. The CrI values of RCNC-H and RCNC-N were 51.9% and 62.6%, respectively [36].

The crystalline structure of RCNC-H and RCNC-N was further investigated by ^13^C solid-state NMR, and the results are presented in Figure 2c,d. The chemical signals of RCNC-H reflected the representative cellulose I structure with peaks assigned to C1 (105.01 ppm), C4 crystalline (89.17 ppm), C4 amorphous (84.50 ppm), C2/C3/C5 (75.01, 72.87, and 71.63 ppm, respectively), C6 crystalline (65.47 ppm), and C6 amorphous (62.79 ppm) [34]. For RCNC-N, a second resonance at 107.39 ppm was also observed for C1, demonstrating cellulose II’s crystal form. In addition, the signal position of C4 crystalline shifted right to 88.93 ppm. This was in accordance with a previous study that reported that the signal position of C4 crystalline shifted from 89.52 ppm to 88.44 ppm after being transformed into cellulose II [36]. The NMR results supported the conclusions of FTIR and XRD tests. The CrI values of RCNC-H and RCNC-N calculated from NMR results were 36.7% and 56.2%, respectively, which were relatively lower than the above-mentioned ones. It is worth noting that the indicator of the amorphous component for the peak height method is substantially influenced by the overlap of crystalline peaks [37], while the CrI values calculated from Equation (2) are approximate and are usually lower than the XRD methods [22]. XRD is more sensitive to the crystalline phase than the amorphous phase, while the NMR calculation does not include all small crystalline peaks [38], but the crystallinity of RCNC-N was higher than that of RCNC-H.

### 3.3. RCNCs Reinforced PVA Films

#### 3.3.1. PVA/RCNC Composite Film Optical Property

**Figure 3:**
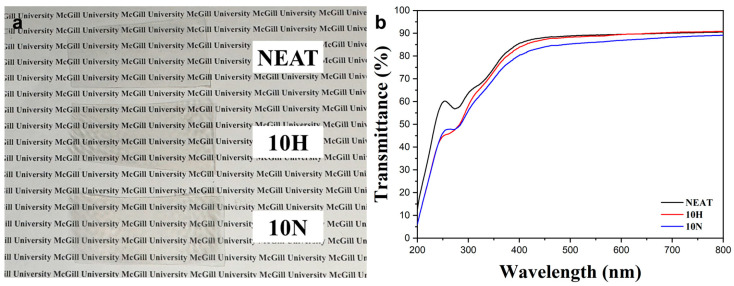
(**a**) Photograph and (**b**) UV-Vis transmittance of PVA/RCNC composite films.

Different amounts (1 wt%, 5 wt%, and 10 wt%) of RCNC-H and RCNC-N were incorporated into the PVA films. It was difficult to see any difference, so only neat PVA, 10% RCNC-H, and 10% RCNC-N films were selected to compare their optical property. As shown in Figure 3a, the PVA film was transparent, and this high transparency was retained after adding 10% RCNC-H or RCNC-N, which might be interpreted by the dimensions of RCNCs and their good dispersion in the PVA matrix.

The UV-Vis transmittance of PVA/RCNC composite films is shown in Figure 3b. The PVA film was capable of blocking a certain amount of UV light and had a transmittance of 90.4% at 800 nm. The addition of RCNCs better blocked the UV light with a transmittance of lower than 50%, while visible light could still easily go through the composite films. The transmittance of 10H and 10N samples at 800 nm was 90.8% and 89.1%, respectively, which were quite similar to that of the PVA film. The high transparency was in accordance with previously reported PVA films that were reinforced by CNCs extracted from okra fibers, where all nanocomposites exhibited a constant transmission level of about 90% at 800 nm [39]. This optical feature is important in food packaging materials used to display food products and can reduce UV exposure.

#### 3.3.2. PVA/RCNC Composite Film Mechanical Properties

**Figure 4:**
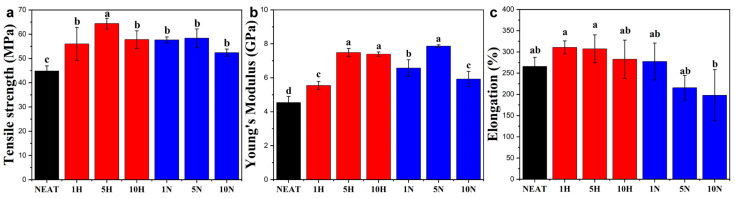
(**a**) Tensile strength, (**b**) Young’s modulus, and (**c**) elongation at the break of PVA/RCNC composite films. Different letters on top of the columns indicate significant differences (*p* < 0.05).

The mechanical properties, including tensile strength, elongation at break, and Young’s modulus of the PVA/RCNC composite films, are shown in Figure 4. Neat PVA films presented a tensile strength of 44.8 MPa and Young’s modulus of 4.5 GPa. The addition of both RCNC-H and RCNC-N significantly improved the tensile strength and Young’s modulus. This could be due to the well-dispersed RCNCs in the PVA matrix that reduced the free space between PVA molecular chains and formed hydrogen bonding interactions with PVA [40]. With the increase in RCNC contents, the tensile strength and Young’s modulus of the composite films first increased and then decreased. When 5% RCNC-H was incorporated, the tensile strength reached a maximum value of 64.4 MPa and was 43.7% higher than that of neat PVA film, while Young’s modulus had an increment of 64.9% with a value of 7.5 GPa. The maximum tensile strength and Young’s modulus of the composite films containing 5% RCNC-N (58.4 MPa and 7.9 GPa) also increased by 30.2% and 73.2%, respectively. The slight drop in the tensile strength at high RCNC loading (10%) might be attributed to the increased brittleness when large amounts of rigid fillers were added [41]. Jahan et al. also reported that the tensile strength of PVA films was proportional to CNC concentrations (0.5–6 wt%) at 53% RH and 93% RH [42], but decreased mechanical properties were observed when more than 2 wt% CNCs (CelluForce NCV100-NAL90) were added to PVA films [43]. This demonstrated the importance of the type of CNCs. Notably, in comparison with RCNC-N, RCNC-H had a slightly better reinforcing effect on PVA films, which was because of the higher aspect ratio of RCNC-H [44]. The needle-like structure of RCNC-H could facilitate the formation of percolating networks for stress transfer in the composites [40,45].

The elongation at the break of PVA/RCNC composite films was not significantly affected by the addition of RCNCs, as shown in Figure 4c. Interestingly, it was noticed that the elongation at the break in the 1H sample rose to 310.6%, and this value slightly decreased when RCNC-H contents increased to 5 and 10 wt%. Similar results have been observed by Fortunati et al. as a consequence of the interfacial bonding between RCNCs and PVA, which allows stress transfer [46]. The incorporation of RCNC-N in PVA resulted in a relatively lower elongation at break, and the value decreased from 265.8% to 197.9% when 10% RCNC-N was added. This might be because the number of RCNC-N particles was more than that of RCNC-H when their contents were the same, which further restricted the motion of PVA molecules during stretching.

#### 3.3.3. PVA/RCNC Composite Film Barrier Properties

**Figure 5:**
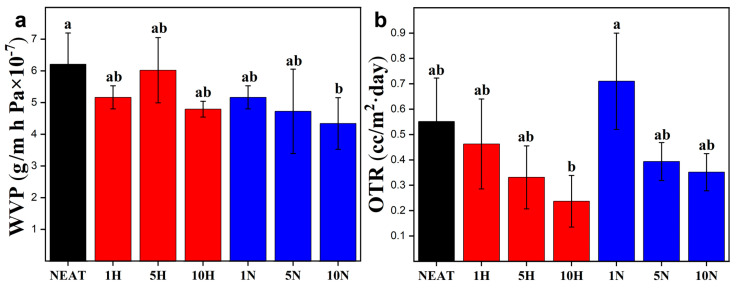
(**a**) WVP and (**b**) OTR of PVA/RCNC composite films. Different letters on top of the columns indicate significant differences (*p* < 0.05).

Materials with low water vapor permeability (WVP) and oxygen transmission rate (OTR) can reduce microbial growth and maintain food quality, which are two crucial features for food packaging applications [47]. As shown in Figure 5, a clear trend was observed in that, with the addition of either RCNC-H or RCNC-N, both WVP and OTR values decreased. PVA films contain a large number of hydroxyl groups, resulting in the hydrophilic feature and a poor water vapor barrier property. The WVP value of neat PVA film was 6.2 × 10^−7^ g/m·h·Pa. It decreased by 16.8% and 30.1% when 10 wt% RCNC-H and RCNC-N were added, respectively, because the dense RCNC nanofillers increased the tortuous pathway within the composite films and slowed down the diffusion of water vapor [48,49]. It was reported that CNCs could act as nucleating sites in the PVA matrix to induce crystallization [50], which might also contribute to the improved barrier properties. Nguyen and Lee declared that the water vapor transmission rate of the PVA film was reduced by 30.3% by adding 5 wt% CNCs, but the dimension of their CNCs (1020 nm in diameter and 300–900 nm in length) was much larger than those of RCNC-H and RCNC-N [51]. Neat PVA film had an OTR value of 0.55 cc/m^2^·day, which declined by 56.4% and 36.4% with the increase in RCNC-H and RCNC-N contents from 0 to 10%. This was because of the reduced free space in PVA/RCNC composite films and the formation of hydrogen bonding interactions between PVA and RCNCs. this type of reduction in OTR was also observed in PVA film containing 30 wt% CNCs (length: 100 ± 25 nm and width: 5 ± 1.5 nm) [52]. It is worth noting that the 10N sample had the lowest WVP value, and the 10H film had the best oxygen barrier property. The former could be attributed to the increased number of RCNC-Ns that better blocked the water vapor [22], and the improved OTR might be due to the larger aspect ratio of RCNC-H that formed the network to tighten the PVA matrix [23].

## 4. Conclusions

In this study, CNCs with different dimensions and crystalline structures were obtained from the same raw material, waste textile, by using two aqueous solvents and acid hydrolysis. The original cellulose crystalline structure was either partially or completely changed during solvent treatments and resulted in RCNC-H and RCNC-N, which had similar diameters but the aspect ratio of RCNC-H was larger than that of RCNC-N. Both could be well-dispersed in PVA films due to their high negative charges. The addition of RCNC-H and RCNC-N improved the UV barrier property of PVA films but did not affect the transparency. It was worth noting that RCNC-H showed relatively better reinforcing effects on tensile strength and oxygen barrier property, and RCNC-N enabled a slightly lower water vapor permeability. Their different dimensions might contribute to these differences: The longer RCNC-H easily formed the percolating network, and the shorter RCNC-N had more nanoparticles at the same concentration. The reinforcing effects of RCNCs on PVA films were generally in accordance with those of previous reports, but some exceptions existed due to the various dimensions of CNCs. Therefore, this initial work demonstrates the possibility to modulate the natural cellulose crystalline structure, and further efforts are expected to explore the relationship between recrystallization conditions and CNC properties.

## Data Availability

Not applicable.
